# Emerging Maripa Hantavirus as a Potential Cause of a Severe Health Threat in French Guiana

**DOI:** 10.4269/ajtmh.22-0390

**Published:** 2023-03-13

**Authors:** Séverine Matheus, Stéphanie Houcke, Guy Roger Lontsi Ngoula, Pablo Lecaros, Jean Marc Pujo, Nicolas Higel, Absetou Ba, Fabrice Cook, Patrick Djahi, Dabor Resiere, Didier Hommel, Anne Lavergne, Hatem Kallel

**Affiliations:** 1 Intensive Care Unit, Cayenne General Hospital, Cayenne, French Guiana;; 2 Emergency Department, Cayenne General Hospital, Cayenne, French Guiana;; 3 Intensive Care Unit, Martinique University Hospital, Martinique;; 4 National Hantavirus Reference Center, Institut Pasteur de la Guyane, Laboratoire Associé, French Guiana;; 5 Tropical Biome and Immunopathology, Université de Guyane, Cayenne, French Guiana

## Abstract

We describe the clinical parameters and management of nine confirmed cases of hantavirus pulmonary syndrome reported in French Guiana since 2008. All patients were admitted to Cayenne Hospital. Seven patients were men and the mean age was 48 years (range, 19–71 years). Two phases characterized the disease. The prodromal phase was characterized by fever (77.8%), myalgia (66.7%), and gastrointestinal symptoms (vomiting and diarrhea; 55.6%) starting, on average, 5 days before the illness phase, which was characterized by respiratory failure in all patients. Five patients died (55.6%) and the length of stay in the intensive care unit was 19 days (range, 11–28 days) for survivors. Detection of two back-to-back recent cases highlights the reason to screen for hantavirus infection during the nonspecific phase of the disease, in particular when concomitant pulmonary infection and digestive disorders are observed. Specific longitudinal serological surveys must also be used to identify other potential clinical forms of the disease in French Guiana.

Hantaviruses are zoonotic negative-strand RNA-envelope viruses belonging to the genus *Orthohantavirus*, family Hantaviridae. In the Americas, they are known as New World hantaviruses and may cause hantavirus pulmonary syndrome (HPS), which can be associated with cardiac failure leading to hantavirus cardiopulmonary syndrome. Since the first recognized human case in the United States in 1993, this disease has become an emerging health concern in Central and South America, with more than 43 genotypes reported.^1^ Nearly half of them are known to be pathogenic for humans. The natural reservoirs are rodents. The virus is present in their urine and excreta, and can infect humans when aerosolized.^1^ The case fatality rate of New World hantavirus varies from 30% to 40%.^2^

The hantaviral diseases are recognized as occurring in two clinical phases: the prodromal phase and the illness phase. The incubation period ranges from 7 to 49 days, depending on the duration of exposure.^3^ The prodromal phase is nonspecific. It includes fever, headache, abdominal pain, myalgia, dry cough, and dyspnea, which generally last for 5 to 6 days. The following illness phase is characterized by a quick progression to respiratory and multiorgan failure requiring intensive care management.^4^

French Guiana, a French overseas territory located on the northern coast of South America, presents climatic and geographic factors favorable to the circulation of a range of zoonotic diseases such as malaria, histoplasmosis, Amazonian toxoplasmosis, Q fever, leptospirosis, yellow fever, Dengue and Mayaro viruses, and others. These human infections can present common initial clinical symptoms that can confuse the initial diagnosis. Furthermore, French Guiana is also favorable to emerging zoonoses. Ecological, environmental, and changes in human demographics and behavior increase the risk of contact with reservoirs, and are the main factors responsible for these emergences.^5^ The identification of the first native human case of hantavirus pulmonary syndrome in 2008 in French Guiana illustrates this phenomenon.^6^

We describe the clinical presentation and the outcome of nine confirmed cases of HPS hospitalized in Cayenne Hospital (the referral hospital in French Guiana) between January 1, 2008 and April 30, 2022. Informed consent was obtained from all patients or relatives and was reported in the medical file of the patient. In addition, at admission, all patients or their relatives received a written information stating their data would be used for research purposes and they had the opportunity to deny use.

Molecular and serological diagnoses and sequencing were performed at the National Hantavirus Reference Center, Laboratoire Associé, Institut Pasteur de Guyane.^6^ All patients tested positive by real-time polymerase chain reaction and IgM serology. Specific Maripa virus infection was confirmed by sequencing.

Patient characteristics and clinical parameters recorded at admission are presented in Table 1. Eight patients were transferred to the intensive care unit (ICU) and the other died in the emergency department. Seven patients were men (77.8%). The mean age was 48 years (range, 19–71 years). All patients were healthy before the disease and all lived on the coastal side of French Guiana, where 90% of the population is located. No patient had a recent history of travel outside the department.

**Table 1:** Epidemiological and clinical parameters of hantavirus respiratory syndrome cases

Parameter	Values	Time of onset,* days; mean (range)
Age, years; mean (range)	48 (19–71)	–
Male gender, *n *(%)	7 (77.8)	–
Medical history, *n *(%)		
Arterial hypertension	2 (22.2)	–
Diabetes	1 (11.1)	–
Coronary disease	1 (11.1)	–
Immunodepression	0 (0)	–
Prodromes, *n *(%)		
All symptoms	–	5 (2–6)
Fever	7 (77.8)	4 (1–6)
Myalgia	6 (66.7)	4 (1–6)
Vomiting	5 (55.6)	3 (1–5)
Diarrhea	4 (44.4)	5 (2–6)
Headache	4 (44.4)	3 (1–6)
Discomfort	3 (33.3)	3 (1–6)
Sweat	3 (33.3)	1 (1–1)
Rhinitis	2 (22.2)	4 (1–6)
Chills	1 (11.1)	1 (1–1)
Parameters at admission		
SAPS II, mean (range)	64 (22–84)	–
SOFA day 1, mean (range)	11 (5–17)	–
Temperature, °C; mean (range)	37.9 (35.1–39.6)	–
Respiratory failure, *n *(%)	9 (100)	–
Crackles, *n *(%)	8 (88.8)	–
Shock, *n *(%)	8 (88.8)	–
Renal failure, *n *(%)	9 (100)	–

SAPS II = Simplified Acute Physiologic Score II, SOFA = sequential organ failure assessment.

* Time before respiratory failure.

During the prodromal phase, the main observed symptoms were fever (77.8%), myalgia (66.7%), and digestive disorders (55.6%) that lasted a mean of 5 days (range, 2–6 days). The illness phase started with dry cough (78%) and dyspnea (66.7%), progressing quickly to respiratory, renal, and hemodynamic failure (Table 1). No patient presented with neurological or hepatic manifestations.

Biologic analyses performed at admission showed renal injury in seven patients, hypoproteinemia in seven, elevated hematocrit in eight, hyperlactatemia in six, elevated C-reactive protein in seven, and normal hepatic and muscular enzymes in all patients (Table 2). Chest X-Ray showed bilateral alveolar infiltrates in all patients and pleural effusion in seven patients. Chest computed tomography was performed in only four patients (44.4%) and showed vessel enlargement, peribronchial cuffing, bilateral Kerley lines, alveolar edema, and pleural effusion. Pleural effusion required drainage in three patients (33.3%). Transthoracic echocardiography was performed in five patients (55.6%) and showed normal left ventricular function. Echocardiographic and hemodynamic investigations were consistent with hypovolemia with hyperpermeability syndrome without cardiac involvement. Eight patients needed mechanical ventilation (MV). All had pink, frothy bronchial aspirates. The remaining patient received high-flow oxygen for 3 days. Patients treated with MV received deep sedation, muscle blockers, norepinephrine, adrenalin (one patient), and dobutamine (one patient). Nitric oxide was administered to four patients without evidence of effectiveness. Two patients were put in a prone position. No patient received fluid infusion or albumin administration. One patient died ∼14 hours after admission to the emergency department and four others died in the ICU, giving a case fatality rate of 55.6%. The length of stay in the ICU was 14 days in survivors treated with MV, and 6 days for the patient who did not receive MV. In the survivors, MV was needed for 4, 20, and 24 days, and renal replacement therapy was used for 5 and 7 days.

**Table 2:** Biologic parameters recorded at admission

Parameter	Normal range	Mean (range)
Urea nitrogen, mmol/L	2.8–7.6	11 (6–18)
Creatinine, μmol/L	80–134	158 (93–196)
Calcium, mmol/L	2.1–2.4	1.7 (1.3–2.2)
Serum protein, g/L	63–84	50 (30–69)
Glycemia, mmol/L	3.9–5.84	10 (1–16)
Albumin, g/L	35–52	24 (18–28)
Sodium, mmol/L	136–145	131 (126–135)
Potassium, mmol/L	3.5–5.1	4.2 (3.3–5.5)
Chlorine, mmol/L	98–107	100 (92–109)
Alkaline reserve, mmol/L	22–29	19 ± 3 (15–22)
Lactate, mmol/L	1.1–2.5	3.8 (1–8)
Magnesium, mmol/L	0.66–1.07	0.7 (0.6–0.9)
Phosphorus, mmol/L	0.87–1.45	1.5 (1–2.1)
Aspartate aminotransferase, UI/L	0–40	28 (10–52)
Alanine aminotransferase, UI/L	0–41	29 (13–58)
Lactase dehydrogenase, UI/L	135–225	614 (367–868)
Bilirubin, μmol/L	0–21	5 (1–8)
Alkaline phosphatase, UI/L	135–225	45 (22–74)
Gamma glutamyl transpeptidase, UI/L	0–60	50 (8–244)
Amylase, UI/L	12–100	89 (19–192)
Creatine kinase, UI/L	39–308	180 (95–262)
Troponin, μg/L	< 0.014	0.086 (0.014–0.250)
Brain natriuretic peptide, ng/L	< 300	5,128 (29–27,535)
C-reactive protein, mg/L	0–5	117 (34–192)
Procalcitonin, μg/L	< 0.05	7 (0.4–25.3)
Leukocytes, Giga/L	4–10	16 (10–23)
Neutrophils, Giga/L	1.5–7.75	13 (8–20)
Lymphocytes, Giga/L	1.0–4.5	1 (1–3)
Platelet count, Giga/L	150–450	101 (50–153)
Hemoglobin, g/dL	13–17	16 (13–21)
Hematocrit, %	42–51	49 (36–67)
Prothrombin, %	70–100	75 (49–94)
Partial thromboplastin time, seconds	0.8–1.2	1.3 (1.1–2.3)
Fibrinogen, g/L	1.6–4	3.6 (2.2–5.3)
D-dimers, ng/mL	0–130	3,266 (481–6,370)

In French Guiana, the first case of hantavirus was detected in 2008 in the framework of an epidemiological survey that started in 2006.^6^ The genome sequence led to the discovery of a new genotype, Maripa virus, related closely to Laguna Negra virus.^7^ Since then, eight other cases have been diagnosed as of this writing. In our study, the prodromal phase started, on average, 5 days before hospitalization, mainly with fever, myalgia, headache, and digestive disorders (vomiting, diarrhea).^2^ Although these symptoms are not specific, the presence of gastrointestinal symptoms can help distinguish hantavirus infection from other causes of pulmonary infection. Thus, in this context, physicians should include hantavirus in the screening for infectious tropical diseases.^8^^,^^9^ In our study, the appearance of dry cough and dyspnea progressed quickly to respiratory failure requiring intensive care, and characterized the beginning of the illness phase. Chest radiography showed bilateral alveolar and interstitial infiltrates related to lesional pulmonary edema. Indeed, hantavirus infects endothelial cells, altering capillary permeability and leading to capillary leak and acute distress syndrome.^10^ Hantavirus infection induces an inflammatory process with a massive release of interleukins and a strong immune response that may be the crucial cause of disruption of the endothelial barrier and organ dysfunction.^4^ The most targeted organs are the lungs and kidneys.^11^ In our study, all patients presented respiratory and renal failure. Eight patients needed MV, seven patients presented hemodynamic failure, and four presented hematological failure. The mechanisms of the hemodynamic and hematological failures are similar to those observed in septic shock.^12^ Unlike data from South America reporting cardiogenic shock in fatal cases of HPS, none of our patients presented cardiac involvement.^2^^,^^4^ All other clinical manifestations are consistent with Brazilian studies.^13^^–^^15^

The management of HPS is symptomatic, and no available and approved etiological therapy was documented. It is based on supplemental oxygen, MV when indicated, fluid balance regulation, and pressors.^16^^–^^18^

In our study, 55.6% of patients died within the first 24 hours of hospitalization. The patient who did not need MV left the ICU after 6 days. The fatality rate is greater than that reported in South America (30–40% for HPS).^2^ The main causes of death are respiratory and hemodynamic failure.^16^ Most deaths occur during the first 48 hours of the clinical course.^13^

Although all cases of hantavirus infection reported in French Guiana were severe, we suspect the presence of potential underdiagnosed mild forms. Indeed, serological surveys showed IgM and IgG reactive to hantavirus in healthy and pauci-symptomatic persons in Peru and Brazil.^19^^,^^20^ In Panama, hantavirus infection was frequent, but resulted rarely in respiratory illness and hospitalization.^21^^,^^22^ To explore this in French Guiana, specific longitudinal serological surveys must be set up for persons with possible exposure to rodents. Close vigilance and large information to first-line physicians are also required. These measures will help identify potential asymptomatic forms of hantavirus in French Guiana.

Maripa virus is an emerging virus in French Guiana. The recent detection of two back-to-back recent human cases suggests that screening for hantavirus infection must be reinforced in patients with concomitant pulmonary infection and gastrointestinal disorders, especially when biologic analyses are concordant with capillary leakage. Specific serological surveys must also be set up to identify potential mild forms of the disease in French Guiana.
